# Combination Therapy of PTH and Antiresorptive Drugs on Osteoporosis: A Review of Treatment Alternatives

**DOI:** 10.3389/fphar.2020.607017

**Published:** 2021-01-27

**Authors:** Chenggui Zhang, Chunli Song

**Affiliations:** ^1^ Department of Orthopedics, Peking University Third Hospital, Beijing, China; ^2^ Beijing Key Laboratory of Spinal Diseases, Beijing, China

**Keywords:** osteoporosis, intermittent parathyroid hormone, alendronate, teriparatide, combination therapy

## Abstract

Antiresorptive drugs have been widely used for osteoporosis. Intermittent parathyroid hormone (PTH), an anabolic agent, increases osteoblast production rate and inhibits apoptosis of osteoblasts, thus increasing skeletal mass besides improving bone microarchitecture and strength. Combination therapy for osteoporosis produced great interests and controversies. Therefore, we performed a systematic literature search from PubMed, EMBASE, Scopus, Web of Science, CINDHL, and the Cochrane Database of Systematic Reviews using the search terms PTH or teriparatide combined with bisphosphonate, alendronate, ibandronate, risedronate, raloxifene, denosumab, and zoledronic acid with the limit osteoporosis. At last, 36 related articles were included for further analysis. Findings from previous studies revealed that combination therapy in different conditions of naive or previous bisphosphonate treatment might have different outcomes. The use of combination therapy, however, may be an alternative option among osteoporotic patients with a history of bisphosphonate use. Combined teriparatide with denosumab appear to show the most substantial and clinically relevant skeletal benefits to osteoporotic patients. Additional research is necessary to define optimal methods of developing sequential and/or cyclical combinations of PTH and antiresorptive agents.

## Introduction

Osteoporosis is the most common metabolic skeletal disorder, characterized by decreased bone mineral density and deterioration of bone microarchitecture, predisposing to increased bone fragility and fracture risk, which increases mortality and disability in both sexes (Compston et al., 2019). With the global population aging, osteoporosis remains a formidable public health problem and an urgent need exists to explore effective therapeutic methods that can fully restore skeletal integrity. Apart from the tissue engineering method such as using nanofiber-based materials, 3D artificial bone and other materials with mechanical strength for improving bone architecture to promote bone regeneration (Sun et al., 2013; Xu et al., 2014; Wu et al., 2020; Zhang et al., 2020a; Zhang et al., 2020b), currently approved medications to treat osteoporosis can be classified as antiresorptive and osteoanabolic. Bisphosphonates as the antiresorptive drugs are first-line anti-osteoporosis drugs for reducing the risk of fracture (Eastell et al., 2003). Bisphosphonates inhibit bone resorption by promoting osteoclast apoptosis, interfering with osteoclast function by acting on osteoclast farnesyl-pyrophosphate synthase, and inhibiting prenylation procedure of small GTPases such as Rabs, Rho, Rac, and Ras (Fisher et al., 1999). Therapy with antiresorptive agents can improve bone strength and reduce the risk of fracture by refilling remodeling space, increasing secondary mineralization, and enhancing bone architecture (Arboleya et al., 2000; Axelsson et al., 2017). However, bisphosphonate therapy can only maintain but not rebuild bone structure when bone mass is lost due to increased bone remodeling in postmenopausal women. Furthermore, the function of bisphosphonate is limited with the occurrence of severe thinning of the trabecular network in patients with severe osteoporosis and high risk of fracture (Kraenzlin and Meier, 2011).

**FIGURE 1:**
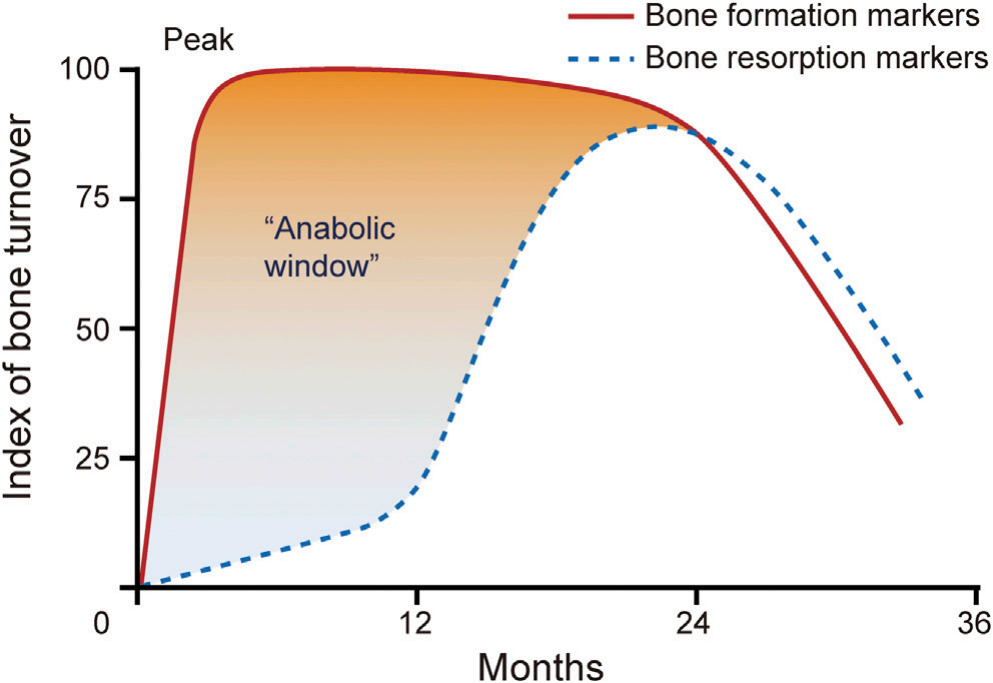
The anabolic window. The figure shows the concept that bone formation is first stimulated by PTH followed by a later increase in bone resorption [adapted from (Pazianas, 2015)].

By now, Teriparatide and abaloparatide are the only two approved osteoanabolic drugs available for treatment of osteoporosis (Tabacco and Bilezkian, 2019; Cheng et al., 2020). The underlying mechanism of the BMD increase is quite different between bisphosphonate and teriparatide (Dempster et al., 2016a). Parathyroid hormone (PTH) acts on PTH receptor 1 and substantially increases bone formation markers (initial bone formation) and later induces an increase in bone resorption markers in a lower degree (later bone remodeling). This sequence of bone formation and resorption leads to an “anabolic window,” which is a period when teriparatide shows maximally anabolic effect (Figure 1; Girotra et al., 2006; Lindsay et al., 2006; Ma et al., 2006; Pazianas, 2015). Increased bone formation by PTH not only increases bone mineral density (BMD) but also improves trabecular number and connectivity; this enhances bone microarchitectural properties and strengthens mechanical resistance to fracture. PTH is recommended when patients are at a high risk of fracture or respond unsatisfactorily to or cannot tolerate other osteoporosis therapies such as bisphosphonates (Hodsman et al., 2005). In clinical settings, patients who fail conventional anti-osteoporosis therapy with bisphosphonates or have recurrent fractures are usually switched to PTH treatment. PTH has also been recommended for the treatment of persistent glucocorticoid-induced osteoporosis (Saag et al., 2007).

However, in animal studies, PTH has been implicated with increasing intracortical bone turnover and porosity during remodeling, which occurs mainly near the endocortical surface (Burr et al., 2001; Mashiba et al., 2001; Sellmeyer et al., 2007). The combined use of PTH with alendronate may reduce the negative effects by preventing PTH-induced increase in cortical porosity (Black et al., 2003). Bone remodeling involves resorption of existing bone by osteoclasts followed by the formation of new bone by osteoblasts. Bisphosphonates inhibit bone resorption by acting on osteoclasts. As a result, bone formation coupled to resorption is also decreased leading to inhibition of bone formation. Meanwhile, PTH increases bone formation with simultaneously increased bone resorption (Martin, 2014). These phenomena interfere with bisphosphonates and PTH therapeutic potentials for osteoporosis. Therefore, combination therapy with an antiresorptive and PTH has been suggested. Combined therapy is supposed to compensate for the limitations and improve the performance of either agent.

However, there is a discrepancy in the effectiveness of combination therapy. While some studies have not found combination therapy to be beneficial than monotherapy in the treatment of osteoporosis (Black, et al., 2003), other studies focusing on bone architecture (de Bakker et al., 2015) have reported the benefits of combination therapy than monotherapy. Although the use of denosumab or bisphosphonate after teriparatide showed benefits in the BMD increase (McClung. 2017), patients receiving antiresorptive drugs accounted for a large portion of those who received PTH treatment due to clinical need (Miller et al., 2008). Combination therapy initiated in naïve patients with osteoporosis or PTH therapy initiated after pre-alendronate monotherapy also significantly influences the outcomes. The outcome of combination treatment is influenced by the treatment-naïve regimen or antiresorptive therapy history. Similarly, whether the antiresorptive agent is continued or stopped when teriparatide is added also affects the results.

Contrasting findings in the efficacy of the combination therapy in the osteoporosis treatment remains unknown. Variation in the dosage, schedule, and administration duration of PTH and bisphosphonate greatly influence the outcomes of combination therapy (Miller, et al., 2008; Shen et al., 2017). Using recently published literature, we aimed to review the effect of using PTH and antiresorptive drugs as a combination therapy for osteoporosis treatment and evaluate the best treatment option.

## Methods

### Search Strategy and Study Selection

A systematic literature search was performed from PubMed, EMBASE, Scopus, Web of Science, CINDHL, and the Cochrane Database of Systematic Reviews using the search terms PTH or teriparatide combined with bisphosphonate, alendronate, risedronate, ibandronate, raloxifene, denosumab, and zoledronic acid with the limit osteoporosis. Further, reference lists of all the articles were hand searched for other relevant publications. Exclusion criteria included articles not relevant to combination therapy, not focus on osteoporosis therapy, non-English articles, conference abstracts, and commentaries. Abstracts of the acquired articles were independently reviewed by two authors (Z. CG, S. CL). After excluding irrelevant articles, the remaining studies were further reviewed by full-text reading to ascertain eligibility.

## Results and Discussion

**FIGURE 2:**
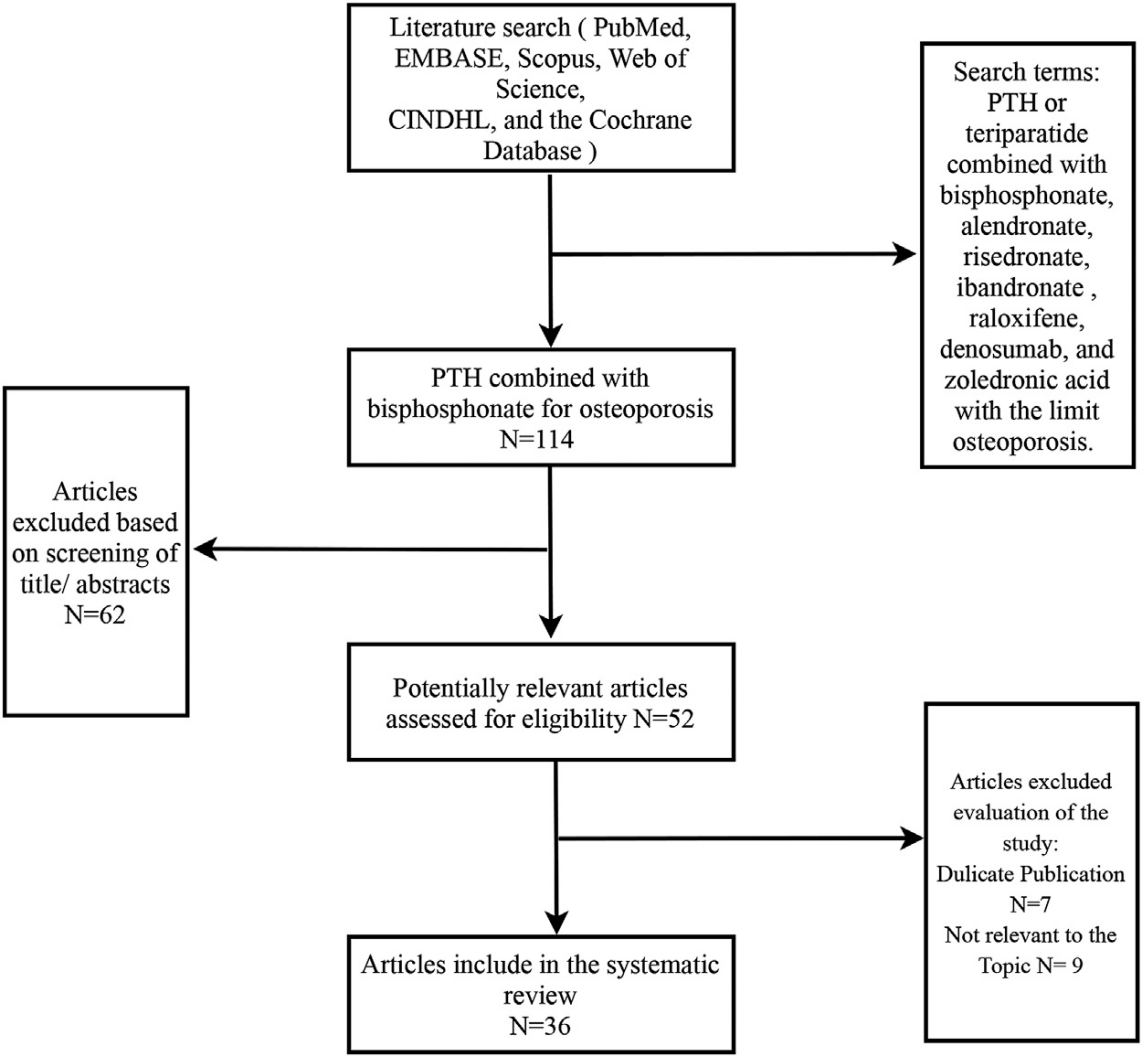
Flow diagram of study selection process. Initially 114 studies were identified. The review process resulted in 36 studies included in this systematic review.

The literature search strategy yielded 100 publications across electronic databases and 14 additional related articles were further obtained by hand searching reference of included studies. A total of 114 articles were retrieved. Finally, 36 related articles (Table 1) were included for further analysis (Figure 2).

**TABLE 1:** Literatures about combination PTH and antiresorptive drugs for osteoporosis.

Journal	Author	Publish year	Bisphosphonate	Trial	Result	PTH dose	Bisphosphonate dose	Main outcome measures	Study duration	Samples
Endocrinology Johnston, et al. (2007)	Sara Johnston	2007	Alendronate	Twenty-week-old female mice	Positive (bone strength and BMD of lumbar vertebra and femur, cortical thickness), low osteocalcin and TRAP	40 μg/kg.d 5 d/wk	20 μg/kg·d 3 d/wk	BMD, bone markers, bone structure, bone strength	7 weeks	70
The New England Journal of Medicine Cosman et al. (2005)	Felicia Cosman	2005	Alendronate	Women with osteoporosis who had been taking alendronate for at least 1 year	Positive (spine BMD both daily and cyclic PTH). NS (hip BMD); high PINP, osteocalcin, BSAP and UN-TCR.	25 μg/daily or three months interval	70 mg alendronate weekly	Bone markers, lumbar spine and hip BMD	15 months	126
Bone Delmas, et al. (1995)	P. D. Delmas	1995	Tiludronate	Old female sheep	NS (osteocalcin, ALP, urinary pyridinoline and hydroxyproline was not different from control). Decrease bone formation and resorption compared with PTH	500 IU/d	1 mg/kg/day	Biochemical and histological indices of bone turnover	3 months	28
The New England Journal of Medicine Finkelstein, et al. (2003)	Joel S. Finkelstein	2003	Alendronate	Men	BMD of lumbar spine and femoral neck and serum alkaline phosphatase increased more with PTH alone. BMD of lumbar spine (combination > alendronate)	40 μg/daily	10 mg/daily	BMD of lumbar spine, femur, radial shaft and total body, serum alkaline phosphatase.	30 months	83
The New England journal of medicine Black, et al. (2003)	Dennis M. Black	2003	Alendronate	Postmenopausal women (who were not using bisphosphonate)	NS (spine BMD between combination and PTH). Volumetric density of the trabecular bone at the spine and bone formation markers were increased more in PTH. Hip and distal one third radius BMD (more in combination and alendronate). Trabecular hip (PTH > combination > alendronate). Decrease of volumetric density of cortical bone and increase of cortical volume in PTH while both increase in alendronate and combination group.	100 μg/daily	10 mg/daily	BMD and quantitative CT of trabecular bone at the spine, femoral neck and hip, bone formation and resorption markers	12 months	238
JBMR Deal, et al. (2005)	Chad Deal	2005	Raloxifene	Postmenopausal women	PINP and spine BMD were increased similarly between PTH (5.19 ± 0.67) alone and combination (6.19 ± 0.65) therapy. Less increase of CTX and larger increase in total hip BMD in combination therapy.	20 ug/daily	60 mg/daily	BMD and biochemical markers of bone turnover and calcium and phosphate	6 months	137
J Clin Endocrinol metab Finkelstein, et al. (2010)	Joel S	2010	Alendronate	Postmenopausal women	Spine, femoral neck BMD, PINP and CTX were increased more in PTH alone	40 μg/daily	10 mg/daily	BMD and bone markers	30 months	93
J Clin Endocrinol Metab Cosman, et al. (2009)	Cosman F	2009	Alendronate raloxifene	Postmenopausal women with osteoporosis on alendronate or raloxifene for at least 18 months	Alendronate: BTM was smaller increased in add group but more increased in total hip and lumbar spine BMD. Raloxifene: BTM was smaller increased in add group and total hip BMD was more increased at 6 months while no significance in total hip, lumbar spine, and femoral neck at 18 months.	20 μg/daily	Alendronate 70 mg/w, raloxifene 60 mg/d	Bone turnover markers (PINP, ALP and βCTX) and BMD	36 months	198
Bone Li et al. (1995)	Li M	1995	Risedronate	Aged ovariectomized rats	PTH induced increase in trabecular width but not number of the lumbar vertebral body, maximum load, and stress. Concurrent treatment with risedronate did not have a greater effect.	80 μg/kg BW 5 days/week	5 μg/kg BW SC 2 days/w	Quantitative bone histomorphometry and biomechanical testing of lumbar spine	10 weeks	120
JBMR Cosman et al. (2011)	Felicia Cosman	2011	Zoledronic acid	Postmenopausal women with osteoporosis	Lumbar spine and total hip BMD were increased more in combination. Combination: PINP increase (0–4 w), decline (4–8 w) then rises. CTX reduce (0–8 w) to a gradual increase.	20 μg	5 mg	BMD and bone markers	1 year	412
JBMR Samadfam et al. (2007)	Rana Samadfam	2007	OPG, alendronate	4-month-old oophorectomized mice	Co-treatment produce additive increase of BMD in femur and supra-additive increase in the lumbar spine than PTH alone. No impact on the mechanical force in PTH.	80 μg/kg/day	Alendronate 100 g/kg/week OPG 10 mg/kg twice/week	BMD and bone strength	2 months	60
JBMR Cosman, et al. (2013)	Felicia Cosman	2013	Alendronate and raloxifene	Postmenopausal women with osteoporosis receiving ALN 70 mg/week (n = 91) or RLX 60 mg/day (n = 77) for ≥18 months	In the alendronate adding group, hip BMD and strength increase more than the switch group while similar increase in spine strength. In the raloxifene adding group, hip BMD and strength increase at 6 and 18 months but only at 18 months in switch group while similar increases in spine BMD and strength. PINP increase more in PTH alone.	20 μg/day	Alendronate 70 mg/week and RLX 60 mg/day	BMD, bone strength and PINP	At least 18 months	182
Bone Yamane, et al., 2009)	Hirotoshi Yamane	2009	Alendronate	Ovariectomized mice	Alendronate enhanced the anabolic actin of PTH at the primary spongiosa (femur, lumbar BMD, BV/TV, Tb. Th, Tb. N of proximal tibia) but blunt it in the remodeling trabecular bone (secondary spongiosa). Osteocalcin and U-Ctx increase more in PTH.	40 μg/kg BW	Alendronate subcutaneous 1 mg/kg BW	BMD, bone volume and bone turnover markers	14 weeks	105
Bone Campbell, et al. (2011)	Graeme M. Campbell	2011	Alendronate	Female retired breeder wistar rats aged 7–9 months.	Combined treatment resulted in more pronounced improvements of the bone architecture than PTH monotherapy (cortical, BV/TV, trabecular thickness, connectivity and stiffness of proximal tibia)	40 μg/kg 5 days/week	15 μg/kg	BMD, synchrotron radiation micro-CT scans *in vivo* and bone strength	14 weeks	40
Bone Altman, et al. (2014)	Allison R. Altman	2014	Alendronate	3-month-old, female, sprague–dawley rats	PTH + ALN treatment had an additive effect (greatest increase in BV/TV and stiffness of tibial trabecular bone), more plate-like not rod-like structure. Bone formation↑Bone resorption↓ PTH + ALN = PTH (Tb.Th)	60 μg/kg/day	50 μg/kg	Bone microarchitecture, bone strength and static and dynamic bone histomorphometry	12 days	30
The journal of biological chemistry Pierroz, et al. (2010)	Dominique D. Pierroz	2010	Alendronate and denosumab	Female huRANKL mice, male RANK^/^mice, and their respective wild-type (WT) littermates	ALN-PTH + ALN > ALN-ALN but not for denosumab (BMD change of total body, lumbar spine and femoral shaft. BV/TV, Tb. Th of vertebral and distal femur trabecular bone. Cortical width) increase of osteocalcin and TRACP5b in PTH + ALN compared with continuing ALN. Osteoclasts are not strictly required for PTH anabolism, which presumably still occurs via stimulation of modeling-based bone formation	80 μg/kg/d	ALN 100 μg/kg 2 times/week denosumab 10 mg/kg 2 times/week	BMD, bone microarchitecture, bone turnover markers	8 weeks	6-8 per groups
Bone Vegger, et al. (2014)	Jens Bay Vegger	2014	Zoledronate	16-week-old female wistar rats	Positive: PTH + ALN (high femoral BV/TV and high Fmax at the distal femur, less Tb.SP ) PTH > PTH + ALN (BFR/BS).	80 μg/kg	100 μg/kg	BMD, bone microarchitecture, dynamic bone histomorphometry and mechanical testing	4 weeks	72
JBMR Cosman, et al. (1998)	Felicia Cosman	1998	Alendronate	Postmenopausal women with osteoporosis already on alendronate 10 mg/day	ALN-PTH + ALN (markers of bone formation increased within 3 weeks in the PTH plus alendronate group, with mean peak levels at 5–7 weeks: OC, PICP, and BSAP. Levels returned to baseline after discontinuing PTH, with PICP declining the most rapidly.)	400 IU/day	10 mg/day	Bone turnover markers	11 weeks	10
Calcif tissue int Arrington, et al. (2010)	Sarah A. Arrington	2010	Zoledronic acid	Female nude mice injected with human breast cancer cells	Supplemental use of PTH did not result in further increase in bone strength but was associated with significant increase in BMD and bone mass of femur.	100 μg/kg daily	25 μg/kg weekly	BMD, trabecular bone volume, and bone strength	12 weeks	56
Journal of bone and mineral metabolism Mashiba, et al. (1995)	TasukuMashiba	1995	Bisphosphonate (cimadronate)	Female sprague-dawley rats aged 11 weeks	Only mid-femoral BMD increased in combination. Concurrent treatment with PTH and bisphosphonate (cimadronate) resulted in a bone anabolic effect not only in cancellous bone but also in cortical bone.	30 μg/kg	5 μg/kg	BMD of distal, middle, and total femur	8 weeks	49
Osteoporos int Li, et al. (2012)	Int Y. F. Li	2012	Bisphosphonate (zoledronic acid)	Osteoporotic rats	No differences in bone mineral density and BV/TV of the contralateral tibiae were observed between treated groups.	60 μg/kg three times a week	1.5 μg/kg/weekly	BMD and BV/TV of tibiae	8 weeks	60
JBMR Ettinger, et al. (2004)	Bruce Ettinger	2004	Raloxifene or alendronate	Postmenopausal women who had previously received either alendronate (ALN) or raloxifene (RLX) therapy for 18–36 months.	Hip BMD (prior ALN -1.8%, RLX +0.5%) and spine BMD (prior ALN +0.5% vs. prior RLX +5.2%) during the first six months and same performance between 6 and 18 months. At 18 months, total hip BMD increased (1.8%, *p* < 0.05) in prior RLX but was not different from baseline in prior ALN. Same with bone turnover marker.	20 μg/daily	ALN 10 mg/day or RLX 60 mg/day	Hip and spine BMD, bone turnover markers	18 months	59
JBMR Dempster, et al. (2016b)	David W Dempster	2016	Alendronate	Postmenopausal women on ALN for at least 1 year still continues ALN or with minimal or no prior osteoporosis therapy	Both Rx-Naive and ALN-RX subjects responded to TPTD with significant increases in bone formation indices at both time points. Within ALN-Rx group, BV/TV and Tb. N were significantly higher, and Tb. Sp was significantly lower in late daily TPTD group. Cortical porosity increased most significantly in late daily TPTD group.	20 mcg/daily or cyclic	70 mg/wk	Bone microarchitecture and bone formation variables from quadruple labels.	7 weeks and 7 months	120
Bone research Nakamura, et al. (2017)	Yukio Nakamura	2017	Denosumab	Primary osteoporotic treatment-naive patients with low L-BMD and/or bilateral hip BMD (H-BMD; less than −3.0 SD).	L-BMD (L1-L4) increased more in combination group at 24 months (17.2% vs. 9.6%).H-BMD tended to be higher in combination group. The combined therapy decreased BAP to a lesser extent than denosumab monotherapy, which may have contributed to the larger BMD increase.	TPTD 20 μg/daily	60 mg once per 6 months	Hip and spine BMD, bone turnover markers.	24 months	30
JBMR Idolazzi, et al. (2016)	L. Idolazzi	2016	Denosumab	Women with severe postmenopausal osteoporosis	Both CTX and P1NP increased in denosumab-TPTD. Hip BMD: Significant changes were observed only with denosumab or combination therapy. No significant differences among treatment groups were observed.	20 mcg/daily	60 mg every 6 months	Hip and spine BMD, bone turnover markers	9 months	59
Lancet Diabetes Endocrinol Tsai, et al. (2019)	Joy N Tsai	2019	Denosumab	Postmenopausal women with osteoporosis	Combined treatment with teriparatide 40 μg and denosumab increased spine and hip BMD more than standard combination therapy.	20 or 40 mcg/daily	60 mg every 6 months	Hip and spine BMD, bone turnover markers	15 months	76
JBMR Cosman, et al. (2020)	Felicia Cosman	2020	Denosumab	Postmenopausal women with osteoporosis	The cyclic regimen did not improve BMD compared with standard regimen at 36 months, however, there appeared to be a benefit at 18 months.	20 mcg/daily cyclic or standard regimens	60 mg every 6 months	Hip and spine BMD, bone turnover markers	36 months	70
JBMR Ramchand, et al. (2020a)	Sabashini K. Ramchand	2020	Denosumab	Postmenopausal women at high risk of fracture	Either high or standard dose of teriparatide improved HRpQCT measures of bone density, microstructure, and estimated strength, along with greater gains in total bone density observed in the HD-group.	20 or 40 mcg/daily	60 mg every 6 months	BMD, microstructure, and strength of distal femur and radius	15 months	69
The Journal of Clinical Endocrinology & Metabolism Leder, et al. (2014)	Benjamin Z	2014	Denosumab	Postmenopausal women aged 45 or older	Lumbar spine, femoral neck and total hip BMD increased more in combination group. CTX and PINP were equally suppressed in denosumab and combination group, where osteocalcin was decreased more in the denosumab group.	20 μg/daily	60 mg every 6 months	Total hip, femoral neck and spine BMD. Bone turnover markers	2 years	100
Endocrine Walker, et al. (2013)	Marcella D. Walker	2013	Risedronate	Men	Combined teriparatide and risedronate increased BMD at the LS, TH as well as the FN and provided greater BMD increase at the TH than monotherapy. PINP and CTX increased rapidly, mirroring the teriparatide alone arm in combined group.	20 μg/daily	35 mg weekly	Total hip, femoral neck and spine BMD. Bone turnover markers	18 months	29
JBMR Muschitz, et al. (2013)	Muschitz C	2013	Alendronate or risedronate	Postmenopausal women with severe osteoporosis on TPTD treatment for 9 months	Except trabecular compartment of femoral neck, lumbar spine BMD, trabecular lumbar spine BMD, total hip BMD, cortical thickness of lumbar spine, femur and hip were increased more in combined ALN group than PTH alone. PINP and CTX decreased and CTX decreased to the starting level in the ALN combination group.	20 μg/daily	ALN 70 mg weekly RAL 60 mg/d	Total hip, femoral neck and spine BMD (cortical and trabecular). Bone turnover markers	18 months	183
J Clin Endocrinol Metab Schafer et al. (2012)	Anne L. Schafer	2012	Ibandronate	Postmenopausal women with low bone mass	PTH + ibandronate 6 months-Ibandronate 18 months vs. PTH 3 months – Ibandronate 9 months. Areal BMD at the spine and hip as well as volumetric BMD were increased similarly. PINP in combined group increased then declined. Sequential treatment increased markedly.	100 μg/daily	150 mg monthly	Hip and spine BMD (cortical and trabecular). Bone turnover markers.	24 months	44
Osteoporos int Cosman et al. (2008)	F. Cosman and J.	2008	Raloxifene	Women with osteoporosis on raloxifene	(Raloxifene- PTH+ raloxifene -raloxifene) vs. (raloxifene- raloxifene- raloxifene) In the PTH + raloxifene group, bone turnover increased 125–584%, spine BMD increased 9.6%, hip BMD increased 1.2–3.6% and radius BMD declined 4.3%. The follow-up year, on continued raloxifene, BMD declined slightly at all sites except the femoral neck.	25 μg/daily	60 mg/day	Total hip, femoral neck, proximal radius, Trochanter and spine BMD. Bone turnover markers	24 months	42
J Clin Endocrinol Metab Boonen et al. (2008)	Steven Boonen	2008	Alendronate, risedronate, etidronate	Women with osteoporosis	Lumbar spine BMD increased at all visits, whereas a transient decrease in hip BMD, which was subsequently reversed. Significant increases in bone formation markers occurred in all groups after 1 month of teriparatide treatment.	20 μg/daily	—	Hip and spine BMD, bone turnover markers	2 years	245
J Clin Endocrinol Metab Tsai et al. (2016)	J. N. Tsai	2016	Denosumab	Postmenopausal osteoporotic women	Trabecular vBMD and cortical thickness increased more in the combination group than both monotherapy group. Radius trabecular vBMD increased more in the combination group.	20 μg/daily	60 mg every 6 months	Radius and tibia BMD.	24 months	94
J Clin Endocrinol Metab Ramchand, et al. (2020b)	Sabashini K. Ramchand	2020	Denosumab	Postmenopausal osteoporotic women	Women treated with the 40 μg/daily regimen achieved clinically meaningful and rapid gains in hip and spine a BMD compared with the 20 μg/daily	40 μg/daily or 20 μg/daily	60 mg months 3 through 15	Total hip, femoral neck and lumbar spine BMD.	15 months	60

### Combination Therapy in Bisphosphonate Naïve Patients May Not Be Beneficial for Osteoporosis Treatment

The effect of the combination therapy in preventing bone loss showed conflicting results in treatment-naïve patients. In some studies, a general combination therapy for osteoporosis was not recommended due to the blunting effect after combining PTH with antiresorptive agents (Delmas et al., 1995).

Some clinical studies did not find this combination to increase the bone mass than either agent. They argued that bisphosphonate reduces the ability of PTH to increase the BMD. The results from either dual-energy x-ray absorptiometry (DXA) or quantitative computed tomography (QCT) demonstrated that PTH alone benefited BMD than when combined with alendronate. In addition, bone turnover markers of the combination therapy arm displayed a suppression curve similar to that of the alendronate alone curve, suggesting that alendronate may have some blunting effect on the ability of PTH to stimulate bone formation (Delmas et al., 1995; Black et al., 2003; Wang et al., 2015).

Combination group (tiludronate co-administered with PTH) compared with the PTH alone group in aged ewes, showed a blunted increase in areal BMD and trabecular and cortical volumetric BMD measured by QCT (Delmas et al., 1995). Ninety-three postmenopausal women with low BMD were divided into three groups including PTH (40 μg/d) alone, alendronate (10 mg/d) alone and alendronate (10 mg/d) and PTH (40 μg/d) combination in a randomized controlled trial (RCT). DXA and QCT demonstrated that the greatest increase of femoral neck and lumbar spine BMD occurred in PTH (40 μg/d) alone group (Finkelstein et al., 2010). Another research covering over 12 months divided naive women into three groups, including PTH 1-84 (100 μg/d) alone, ALN (10 mg/d) alone, and PTH 1-84 (100 μg/d) + Alendronate (ALN) (10 mg/d). PTH alone group showed a typical increase in BMD. However, a severe blunting effect by alendronate on PTH in increasing BMD was observed using QCT. Similar results were observed in the bone turnover markers including PTNP (procollagen I N-terminal peptide) and CTX-I (C-terminal telopeptide of type I collagen) (Black, et al., 2003). Besides women, 83 men with osteoporosis were randomized to teriparatide (TPTD) at (40 μg/d) alone, ALN (10 mg/d) alone, TPTD (40 μg/d) followed by 6 months of ALN (10 mg/d) pretreatment, and TPTD (40 μg/d) combination with ALN (10 mg/d) for 2 years therapy. The TPTD alone group (18.1%) showed the most increase in spine BMD than the combination group (14.8%) and ALN alone group (7.9%). The lateral spine and femoral neck showed similar trends (Finkelstein et al., 2003).

Schafer et al. combined PTH (1-84) (100 μg/d) and monthly IBN (150 mg/month) therapy using two different dosing regimens. 1) Combination therapy: combination with PTH and IBN for 6 months, followed by 18 months of IBN alone. 2) Sequential therapy: PTH alone for 3 months followed by 9 months of IBN for two sequential courses. Using QCT, the volumetric BMD of the hip in the sequential therapy showed a higher volumetric BMD after two years. In the first few months, concurrent therapy resulted in a rapid increase in the bone turnover marker, followed by a gradual decrease after 2 years. Markers increased to a maximal value at 3–4 months in the sequential therapy group, followed by a gradual decrease when IBN was used alone. However, in the second 3-months PTH therapy, the bone turnover marker increased again though the increase was inferior to the first course, perhaps benefiting BMD (Schafer et al., 2012). During fracture healing, alendronate combined with PTH showed no difference in bone mineral density and BV/TV of the contralateral tibiae compared with the other groups (Li et al., 2012).

Areal bone density, presumably believed to be an indicator of bone strength and better anti-fracture efficacy, was prone to be blunted in the combination group. However, strength cannot be predicted by changes in areal BMD alone because antiresorptive and anabolic agents exert different traits such as magnitude and the direction of the matrix volume or mineral content. Femoral strength was evaluated in four different groups: 1) PTH switch to ALN; 2) Combination switch to ALN; 3) ALN switch to ALN; and 4) PTH switch to Placebo. Nonlinear finite element analysis of the QCT examinations was used. After 1 year, PTH (mean, 2.08%) and ALN (3.60%) significantly changed the femoral strength compared with the other groups. After 2 years, there was a significant increase in the femoral strength in PTH switch to ALN (7.74%), Combination switch to ALN (4.18%), and ALN switch to ALN (4.83%) treatment groups but not for PTH switch to Placebo (1.17%). However, the combination treatment did not show the most effective outcome (Keaveny et al., 2008).

. Overall, based on the findings from the above studies, combination therapy may not to an alternative option for osteoporosis therapy due to a lack of additive effects. However, as noted in these studies, data focusing on the reduction of the risk of the fracture using combination therapy are largely lacking.

### Potential Mechanism Attenuating Combination Therapy

The ability of bisphosphonate to reach the lacuna-canalicular system and restrict the function of osteocyte is suggested to result in the blunt effect. The matrix embedded by bisphosphonate may interact with the first responding bone cells, the bone lining cells, through an unknown physicochemical mechanism. Alendronate pre-treatment could perhaps have blunted the osteocytes less sensitive to the anabolic function of PTH (Tsuboi et al., 2016). Besides, changes in the composition of bone material and microstructure are unlikely to occur and efficacy could not be determined. Quantification of the effects of the combination therapy based on bone material composition, microarchitecture, and strength could be different from the BMD outcome (Seeman and Martin, 2015).

### Combination Therapy is Beneficial for Osteoporosis Treatment

Some studies found a significant increase in BMD or improvement in microarchitecture after using the combination therapy (Cosman et al., 1998; Ma et al., 2003; Samadfam et al., 2007). PTH alone, ibandronate alone, and the combination treatments were all efficient in partially reversing the ovariectomized osteoporosis. Among the three groups, the combination therapy group showed more benefit in mean BMD. This group had an added advantage in preserving cortical bone geometry because of enhanced periosteal formation by PTH and reduced endocortical resorption by ibandronate. Combination therapy also significantly increased collagen formation and decreased its degradation. In addition, it significantly increased viscosity, which was accompanied by a denser alignment of collagen fibers and hydroxyapatite crystals with fewer pores (Yang et al., 2013). In a previous report, PTH (1-34) (30 μg/kg) three times per week combined with bisphosphonate cimadronate (YM-175) (5 μg/kg) showed a bone anabolic effect in both the cancellous bone and the cortical bone. Only the combination group showed an increase in both the mid-femoral BMD and the distal femoral BMD like the PTH alone therapy, suggesting an absence of a blunt effect (Zhang et al., 1998). Another study showed that a combination of PTH with risedronate could improve bone formation in the mandible, indicating the potential advantages of combination therapies in oral disease (Hunziker et al., 2000). Combination risedronate and teriparatide therapy shows promise as a treatment for osteoporosis (Walker et al., 2013). However, the response of different spongiosa to PTH in BMD change showed different outcomes. In the primary spongiosa without remodeling bone, the osteoblast activity closes to the erosion zone did not depend on the presence of the osteoclast. The erosion zone at the chondro-osseous junction is a site of vascular invasion into the growth cartilage plate, which can produce many factors. In the secondary spongiosa with remodeling bone, the coupling between bone formation and bone resorption was evident (Yamane et al., 2009). A 6 months randomized, double-blind trial investigating the effect of teriparatide plus raloxifene combination therapy showed that increased bone formation in combination group was similar as teriparatide alone, however, the increase in bone resorption was significantly less and total hip BMD was significantly increased in combination therapy compared with teriparatide alone (Deal et al., 2005). Compared with PTH, co-treatment produced additive increase of BMD in femur and supra-additive increase in the lumbar spine in combination treatment with OPG or alendronate and PTH (Samadfam, et al., 2007). A meta-analysis of randomized controlled trials also suggested that compared with anabolic agents alone, the combination of anabolic agents and bisphosphonates achieved more gains in the BMD at the total hip and femoral neck within a shorter term (6–12 months) and showed similar benefits on BMD for the longer term (18–24 months) (Lou et al., 2018).

A 1 year partially double-blinded RCT was launched with zoledronic acid (ZOL) (5 mg) dose once yearly plus TPTD (20 μg) injections daily as the combination group. Compared with the zoledronic acid alone and PTH alone groups, the combination group showed a positive outcome. After 52 weeks, 2.3, 1.1, and 2.2% increment in the total hip BMD and 7.5, 7.0, and 4.4% increment in lumbar spine BMD were discovered in the combination, PTH and zoledronic acid groups, respectively. It was concluded that although PTH alone increased spine BMD more than zoledronic alone and zoledronic acid alone increased hip BMD more than PTH alone, PTH combined with zoledronic acid could have an additive effect and a substantial increment in BMD (Cosman et al., 2011). It was further suggested that bisphosphonates did not blunt the anabolic response to PTH and early osteoblastic effect of PTH was independent of the new bone remodeling (Cosman et al., 2011). On the contrary, adding zoledronic acid to PTH could further improve the cortex by preventing PTH-induced increase in cortical porosity (Cosman, et al., 2011). However, although BMD increased in the first 6 months, unfortunately, there was less increase in the second 6 months, hence no net increase in BMD was finally registered. An initial inhibition of the bone turnover marker was found in the combination group with a subsequent increase (Cosman, et al., 2011).

Denosumab, the antibody that neutralizes RANKL, is one of the most promising new drugs for the treatment of osteoporosis (Fassio et al., 2017). Denosumab combined with PTH had additive effects on areal BMD and beneficial effects on the bone microarchitecture (Leder et al., 2014; Nakamura et al., 2017). Compared with individual treatments, combined teriparatide with denosumab after two years had improved bone microarchitecture and increased estimated strength, particular in cortical bone (Tsai et al., 2016). A randomized controlled phase 4 trial showed that spine and hip BMD was increased in combined treatment with teriparatide 40 μg and denosumab compared with standard combination therapy (Tsai et al., 2019). Both short-term treatments combining denosumab with either high (40 μg) or standard (20 μg) dose teriparatide were identified to improves HR-pQCT measures of bone density, microarchitecture, and estimated bone strength (Ramchand et al., 2020a). Another study also indicated that treatment with the HD regimen (40 μg) showed clinically meaningful and rapid increase in hip and spine BMD compared with the SD regimen (20 μg) (Ramchand et al., 2020b). The cyclic regimen of teriparatide combined with denosumab appeared to be beneficial in improving BMD at 18 months, especially in the highly cortical skeletal sites, which was clinically relevant in patients at high imminent risk of fracture, particularly at nonvertebral sites (Cosman et al., 2020). Combination therapy with PTH and denosumab or ZOL seemed to achieve higher BMD gains compared to each agent alone (Leder, 2018; Kelly and Garapati, 2019; Anastasilakis et al., 2020). Mid-femoral BMD was only increased in concurrent treatment with PTH and cimadronate, also suggesting combination result in a bone anabolic effect not only in cancellous bone but also in cortical bone (Mashiba et al., 1995).

Combination therapy of PTH and alendronate has been shown to increase bone volume fraction and plate-like trabecular microstructure than either monotherapy. It was concluded that through rod connection and filling of plate perforation, bone stiffness increased more efficiently and rapidly in the combination therapy than in either monotherapy (Campbell et al., 2011). A similar improvement on the stimulation of new bone formation and trabecular thickness was observed in the combination treatment and PTH alone therapy. The relative bone volume and number of plate-versus rod-like structures were also improved after ALN addition. Although no mechanical outcome was yielded, the majority of the trabecular plates are axially aligned with the primary orientation of daily load bearing. Most trabecular rods horizontally connect trabecular plates to stabilize the whole bone structure. Trabecular plates are often regarded as the most critical determinant factors of bone strength. This is because the plate-like trabecular bone volume is the sensitive predictor of trabecular bone’s elastic moduli and yields strength (Altman et al., 2014).

The greater the loss of the trabecular number and connectivity, the less the efficacy of the treatment in restoring the mechanical properties of trabecular bone. Therefore, to benefit from pharmaceutical treatment, it is critical to treat patients before a significant loss of the trabecular number and connectivity (Guo and Kim, 2002). Therefore, combination therapy may be more favorable in this aspect. The effects of the combination of alendronate and teriparatide in ovariectomized rats were examined in a previous study. Interestingly, the combination therapy group showed a recovery of perforations without an occurrence of trabecular tunneling and cortical porosity. The combination group increased trabecular thickness and number as well as filling holes in plate-like elements. The existing trabecular structures were finally thickened and maintained connectivity after 8 weeks (Campbell, et al., 2011; Altman et al., 2015). A systematic review and meta-analysis also indicated a better improvement of the lumbar spine and total hip BMD was achieved in combination therapy without risk of serious adverse events compared with monotherapy (Lou et al., 2019).

### Anabolic Function of PTH was not Influenced in Absence or Inhibition of Osteoclast Cell

Despite extensive pretreatment with alendronate, 17α-ethinyl estradiol or raloxifene, the mature skeleton of OVX rats remains highly responsive to teriparatide (Ma, et al., 2003). Although prior treatment with ALN prevents increase in BMD, previous treatment with raloxifene achieved an expected teriparatide-induced BMD increase (Ettinger et al., 2004). Ten postmenopausal women with osteoporosis were divided into two groups: the ALN group and the combination group (ALN and PTH). Bone formation markers were increased in the combination group within 3 weeks, with a mean peak level at 5–7 weeks (osteocalcin 49%, propertide of type I procollagen 61%, and bone-specific alkaline phosphatase 24%). PTH can stimulate bone formation and increase bone formation markers in the presence of potent bisphosphonate (Cosman, et al., 1998). Patients who had received long-term alendronate and those who were treatment naïve both had increased cortical bone formation and cortical turnover after 24 months of teriparatide treatment (Ma et al., 2014). In addition, combination therapy of PTH with bisphosphonate was once identified to improve bone quality by repairing microdamage that could result in a fracture if untreated (Ettinger et al., 2013). Even when bone turnover was completely suppressed by denosumab treatment, teriparatide could express its biological activity, resulting in the adjunctive positive BMD effects of the combination therapy (Idolazzi et al., 2016). In addition, despite previous long-term exposure to antiresorptive therapies, Teriparatide could induce positive effects on BMD and markers of bone formation in postmenopausal osteoporosis women (Boonen et al., 2008).

**FIGURE 3:**
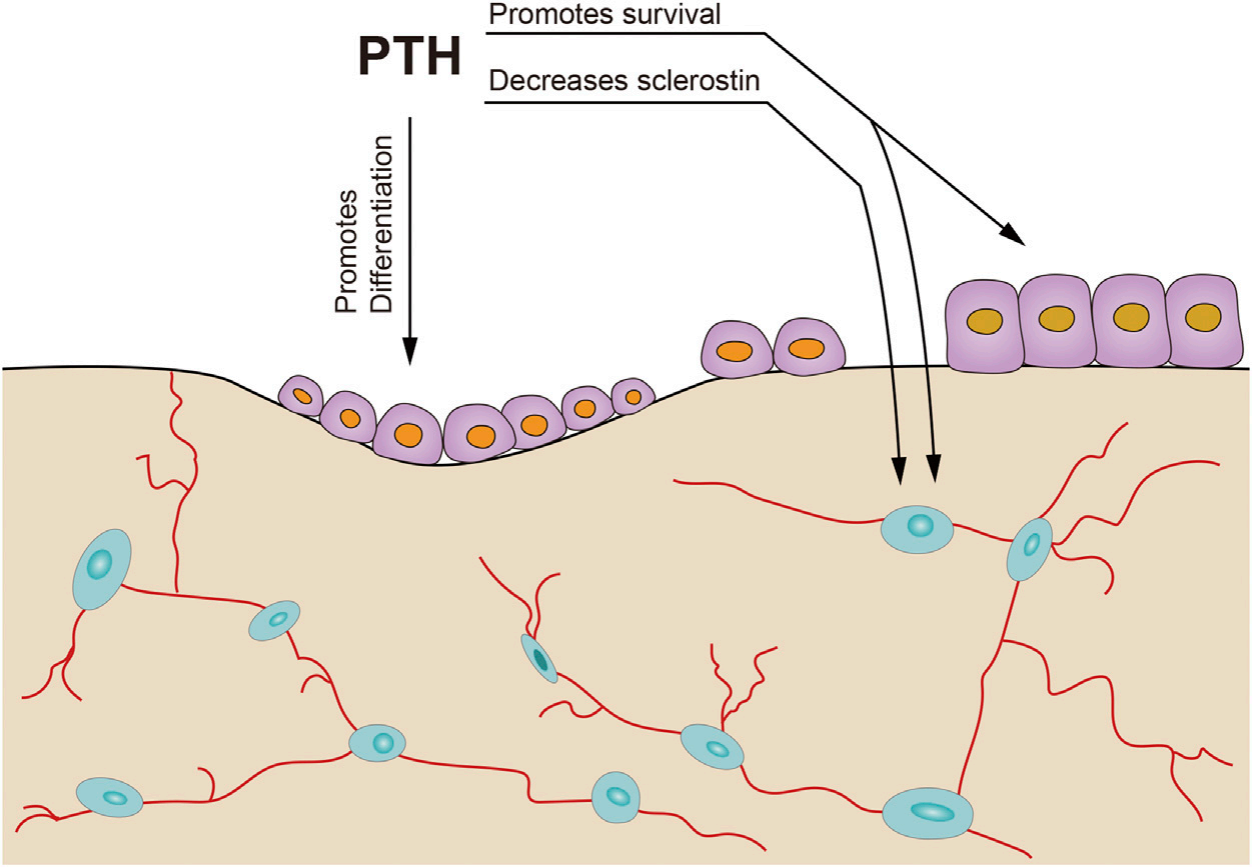
Possible mechanisms of PTH anabolic action without prior osteoclast resorption. PTH could inhibit sclerostin production by osteocytes, promote survival of osteocytes and osteoblasts, and accelerate osteoblast differentiation in partly filled basic multicellular units [adapted from (Martin, 2014)].

Ovariectomized hu-RANKL knock-in mice were first interfered with using a human RANKL inhibitor denosumab, alendronate, or vehicle, respectively for 4 weeks, followed by 4 weeks of combination with intermittent PTH. The addition of PTH to ALN showed improvement in bone mass and microstructure when compared to denosumab. They further applied a massive dose of PTH to RANK-null mouse for two weeks and discovered an elevation in bone formation marker and trabecular bone volume fraction (BV/TV). This indicated that osteoclasts might not be necessary for the anabolic actions of PTH (Figure 3; Pierroz et al., 2010).

A total of 125 postmenopausal women with severe osteoporosis on TPTD treatment for 9 months were divided into three groups for another 9 months: ALN (70 mg/week) in addition to TPTD (for fear that increased resorption could outweigh the bone anabolic effect of the long-time use of PTH); RAL (60 mg/d) in addition to TPTD; or no intervention in addition to TPTD. Although no significant difference was found in the trabecular compartment of the femoral neck, alendronate was suggested to reopen the anabolic window of PTH. This could exert a more robust increase in BMD in other bone areas such as the lumbar spine (5 ± 6.3% ALN combination therapy and 2.8 ± 9.3% PTH monotherapy) and the total hip BMD (4 ± 5.3% ALN combination therapy and 1.4 ± 5.1% PTH monotherapy), as well as trabecular and cortical bone sites when evaluated by 3D CT. While PTNP and CTX concentrations did not change in the PTH alone group, there was a decrease in the ALN combination group and the CTX concentration decreased to the initial level. This implied that ALN assisted the restoration of bone resorption to levels comparable to the initial use of PTH, whereas bone formation remained less suppressed and even surprisingly increased (Muschitz et al., 2013).

To quantitatively track the process of long-bone trabecular remodeling for bone microstructure change, different advanced imaging techniques were used. Early response of bone resorption and formation after combination therapy (PTH and alendronate) was determined using micro-computed tomography (μCT)-based, three-dimensional (3D) *in vivo* dynamic bone histomorphometry technique with a function of longitudinally and simultaneously quantifying changes in bone formation and resorption in rat tibia. A total of 29 rats were randomized into four treatment groups: PTH (PTH 60 μg/kg/d, n = 10), alendronate (ALN 50 μg/kg every three days, n = 6), combination with PTH and ALN (PTH + ALN, n = 6), or vehicle (vehicle, n = 7). The combination treatment group showed both elevated bone formation and low resorption. Consistent with traditional static and dynamic histology, greater bone formation rate per bone surface (BFR/BS) (602 and 514%) and relatively lower bone resorption rate per bone surface (BRR/BS) (82 and 76%) were shown in PTH + ALN group. A similar corresponding increase was also seen in the trabecular bone volume fraction (BV/TV), trabecular stiffness, mineral apposition rate (MAR), and mineralizing surface per bone surface (MS/BS). Overall, high bone formation rate and concurrent low bone resorption rate in the PTH + ALN treated rats indicate that the combination therapy simultaneously has an additive effect on bone modeling activation and bone remodeling inhibition (de Bakker et al., 2015). It was suggested that PTH could increase modeling-based bone formation even in the presence of bisphosphonate (bone formation in absence of resorption), thus concurrent PTH and alendronate may active new bone formation and further inhibit bone resorption, achieving an improved bone quality (de Bakker et al., 2015).

**FIGURE 4:**
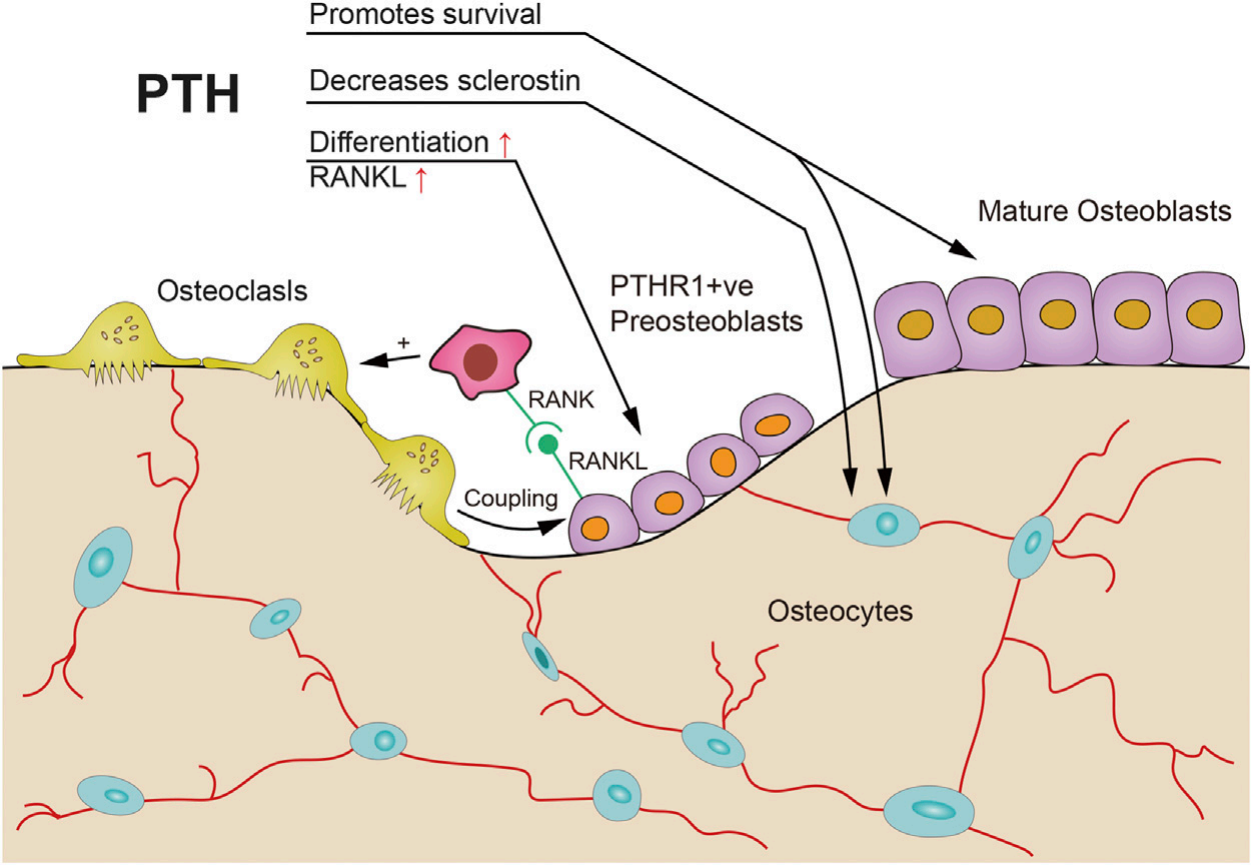
Possible mechanisms of PTH anabolic action with prior osteoclast resorption. PTH could promote survival of osteoblasts and osteocytes, promote differentiation of osteoblast precursors, activation of osteoclasts that induce coupling activities, and inhibit sclerostin production by osteocytes [adapted from (Martin, 2014)].

Concerning osteoblast and osteoclast coupling mechanism (Figure 4), inhibition of osteoclastic release of a negative regulator in osteoblastic differentiation or suppression of positive regulators of osteoblast proliferation may occur. Also, the direct release of osteoblast modulators from osteoclasts was speculated (Ma et al., 2003). However, some authors argue that the observed increased benefit of PTH combination with anti-catabolic therapy in both the lumbar spine and femur could be due to the inhibition of PTH related bone resorption; and the increased efficacy of the combination therapy may at least in part retard the achievement of an equilibrium bone turnover caused by PTH (Ma et al., 2003). Therefore, rather than impairing the anabolic actions of PTH, inhibition of osteoclastic activity may have been beneficial for the efficacy of PTH (Ma et al., 2003). Anyway, the overall benefit of using an anti-remodeling agent might outweigh the drawbacks of a relatively small delay in the anabolic action and a small reduction in bone mass (Ma et al., 2003).

### Osteoporosis with a History of Bisphosphonate Use

Long-term use of bisphosphonate has been associated with a hip fracture or unsatisfactory outcome of bisphosphonate therapy, thus warranting treatment alternatives. Patients who suffer hip fractures or experience unsatisfactory outcome of bisphosphonate therapy due to long-term bisphosphonate use accounted for a large population. At least 50% of all PTH treatments were initiated based on previous treatment of antiresorptive agents, mainly bisphosphate (Cosman, 2014a). Naïve bisphosphonate treatment patients often differ from those with a bisphosphonate treatment history. Compared with bisphosphonate naïve patients, patients established on antiresorptive therapy for osteoporosis have significant differences in the amount of bone surface undergoing remodeling and dynamic changes in endogenous PTH function, which may alter their response to PTH treatment (Cosman, 2014a).

However, several studies indicated that during osteoporosis treatment, prior antiresorptive treatment did not differ from naïve antiresorptive treatment. Thus, even after prior long-term exposure to antiresorptive therapies, PTH treatment was still effective in increasing bone formation, BMD, and bone strength. Prior evidence indicated that previous antiresorptive therapy duration and the time between the stoppage of the previous therapy and commencement of teriparatide did not influence BMD response at any skeletal site (Boonen et al., 2008). Two studies found that adding teriparatide to on-going alendronate could have a superior effect on BMD than using alendronate alone. This implied that, although the absolute increase of bone markers was lower with the added regimen, the anabolic window (the difference between increases in bone formation over bone resorption) was greater in the add regimen (Finkelstein et al., 2003). Substantial gains in BMD of the spine and hip are observed with one year of PTH treatment in postmenopausal women with osteoporosis on raloxifene (Cosman et al., 2008). In addition, nonlinear finite element analysis showed that significant increase in strength compared with baseline was seen in the add group in both alendronate and raloxifene treated postmenopausal women (Cosman et al., 2013).

Meanwhile, other studies evaluated the effects of PTH following osteoclasts inhibition or absence due to treatment with bisphosphonates. In all the studies, anabolic responses of PTH were still observed (Johnston et al., 2007). Taken together, these studies suggested that PTH could induce bone formation without prior osteoclast resorption in both rodent and clinical models (Figure 3). Adding either cyclic or daily TPTD to ongoing ALN treatment may be an effective therapy for patients with severe osteoporosis already treated with ALN who remain at a high risk of fracture (Dempster et al., 2016b).

### Combination Therapy for Other Osteoporotic Conditions

Combined therapy can also be applied in other fields. For instance, the combination treatment in reducing fracture risk after radiation therapy was introduced. Nude mice were strictly divided into four groups: no treatment, radiation, radiation plus Zoledronic acid (ZA) (4–12 weeks), and radiation plus ZA (4–8weeks) and PTH (8–12 weeks) (Arrington et al., 2010). The results indicated that ZA could prevent and reduce the risk of bone fragility and the addition of PTH could increase BMD and bone mass although bone strength was not achieved. Additionally, PTH was suggested to participate in potential anabolic strategies to influence bone remodeling after radiation therapy (Arrington et al., 2010). A combination of alendronate and anabolic drugs has also shown significantly added benefits in osteopenia female rats (Vegger et al., 2014).

### Switch or Add PTH Dilemma

In women treated with long-term antiresorptive agents, response to PTH differed depending on whether the prior antiresorptive agent was continued or stopped. The effect of PTH varies in patients who have received recent treatment with potent antiresorptive (Cusano and Bilezikian, 2012). In many regions, anabolic treatment is recommended when the level of BMD falls or suffers from fracture whilst bisphosphonate treatment is recommended as a rescue treatment. The majority of patients switching or adding to PTH had been previously treated with antire-sorptives (Cosman, 2014a). However, optimal treatment is not attained after long-term bisphosphonate treatment.

### A “Switch to PTH” Choice May not be Good Compared with Combination Therapy

Research showed that when individuals who previously used potent bisphosphonates are switched to TPTD, hip BMD declines below baseline value in the first 12 months after switching. If the antiresorptive drug used is denosumab, the transient hip BMD loss is even more pronounced. This may cause a decline in hip strength during this switching period thereby increasing susceptibility to hip fracture. Moreover, the effects of bone loss at the hip region are more dramatic than for the spine. Although slightly blunted, effects of TPTD after bisphosphonates and denosumab in the spine BMD are still positive (Leder et al., 2015). In addition, in women with osteoporosis on alendronate or raloxifene for at least 18 months, greater increases of bone turnover were achieved by switching to teriparatide, whereas greater increases of BMD were obtained by adding teriparatide (Cosman et al., 2009). The differences in BMD across groups may be due to a relatively greater opening of remodeling space in Switch vs. Add groups. It is likely that the sharp withdrawal of bisphosphonate may produce an exaggerated bone resorption response to PTH, particularly in cortical bone incorporating low amount of bisphosphonate, causing a hip BMD decline in the first 6 months, consistent with the switching of bisphosphonate to PTH monotherapy. Prior denosumab treatment produced marked bone resorption in the cortical bone in a manner that bone formation could not compensate, resulting in decline in hip BMD. This phenomenon was partly identified by the previous research result which show that giving a single intravenous infusion of ZOL at 6 months after the last denosumab injection could prevent bone loss for at least 2 years independently of bone turnover rate (Anastasilakis et al., 2019). Although bone resorption also increased rapidly in cancellous bone, TPTD produced more rapid bone formation, leading to only a very minor transient loss that can be quickly reversed. This indicates that combination therapy was effective and secondary mineralization might be greater in Add than in the Switch groups (Cosman, 2014a). The decision of switching to teriparatide for patients who use long-term bisphosphonate for anti-osteoporosis should be made with caution, especially for those who are at a high risk of hip fracture (Lyu et al., 2019).

Which situation could be suitable for combination therapy? In the above switching condition, it has been shown that with addition of PTH, continuing other than stopping antiresorptive agents increased more in spine and hip BMD (Cosman et al., 2013). Some studies reported that osteoporosis patients treated with bisphosphate sustained a hip fracture or suffered a continuing decline in BMD. They recommended TPTD for such patients than bisphosphonate monotherapy or TPTD monotherapy. Patients with multiple prior fractures due to severe osteoporosis in naïve condition or with history of bisphosphonates treatment were all recommended for combination treatment for improved bone mass and bone strength (Cosman, 2014b).

Recently, Fahrleitner-Pammer and colleagues reported improvements in cancellous bone volume, although a nominally superior response was observed in the treatment-naïve group. Collectively, prior studies indicated that TPTD effectively promoted cancellous bone formation and improved mass and structure both in treatment-naïve and alendronate-pretreated patients (Fahrleitner-Pammer et al., 2016). However, administration of TPTD to patients receiving alendronate could prevent the increase in cortical porosity seen in individuals switched from alendronate to TPTD and balance the formation vs. resorption (Cosman et al., 2017). Sequentially administration of denosumab with teriparatide in combination for 24 months showed great benefits for BMD, which may be a reasonable option for patients with hip fracture risk (Anagnostis et al., 2019).

### Advantage of Combination Therapy for Osteoporosis (Possible Mechanism Explanation)

Selye first reported that in 1932 that a small dose of PTH stimulated osteoblasts and bone apposition without a previous osteoclast formation. On this basis, the first concept of bone modeling was proposed. Lindsay subsequently demonstrated that quadruple tetracycline labeling stimulated bone modeling at trabecular bone. Bone modeling refers to a process in which bone reshapes due to independent action of osteoblast and osteoclast. Generally, bone modeling is common during skeletal development and growth period although it occurs throughout life. Remodeling due to bone formation leads to periosteal expansion while remodeling caused by resorption is responsible for the medullary expansion in the long bones. Bone mass response to some osteoporosis treatments in humans suggests non-remodeling mechanisms play an important role and perhaps bone modeling is the main influence factor. Bone in the PTH group displays a 20–30% bone modeling (Figure 3) and bone formation were all bone remodeling (Figure 4) in the control group (Lindsay et al., 2006; Anagnostis et al., 2019).

Antiresorptive agents use is permissive for modeling at cortical cortex that the function of PTH is not restricted. In addition, antiresorptive drugs inhibits the recruitment of osteoclasts and decrease remodeling, leading an early increase in bone mass (Langdahl et al., 2016). In summary, a combination of PTH and antiresorptive agents improve bone regeneration by controlling modeling and improving secondary mineralization. Antiresorptive therapy can reduce the number of basic multicellular units (BMUs) in the remodeled bone and decrease bone volume in each BMU resorbed. In comparison, PTH creates more bone by increasing the number, volume, and activity of new BMUs in the remodeling bone, and correcting the negative BMU balance (Seeman and Martin, 2015).

Bisphosphonate exerts negative effect on the bone as it increases microcrack density due to reduced matrix ductility as a function of increased collagen cross-linking by pentosidine and other advanced glycation product (AGEs). In addition to decreasing bone resorption, smaller osteons were formed. The role of PTH co-administration can be explained as an ability of replacing highly mineralized and glycated bone generated by the deleterious effects of long-term use of antiresorptive drugs with new osteoid. Co-administration of PTH therapy resulted in overfilled cavity and increased the rate of remodeling, contributing to a focal reconstruction of the skeleton (Seeman and Martin, 2015).

PTH can active the flattened osteoblast lineage cells in the quiescent bone surface of bone periosteal and endosteal surfaces, differentiation, maturation and longevity of all those cells (Figures 3, 4). In other words, PTH works by directly acting on osteoblast lineage cells thereby increase differentiation of committed precursors while inhibiting osteoblasts and osteocytes apoptosis (Seeman and Martin, 2015). However, the possible mechanisms of combination for osteoporosis needs to be further explored in different aspects.

## Conclusion

Combination therapy in different conditions of naive or previous bisphosphonate treatment might have different outcomes. The use of combination therapy, however, may be an alternative option among osteoporotic patients with a history of bisphosphonate use. Combined teriparatide with denosumab appear to show the most substantial and clinically relevant skeletal benefits to osteoporotic patients. Although addition of PTH appears to be advantage than switching to PTH, any possible benefits of combination therapy must be weighed in relation to costs and inconveniency caused by taking two drugs as opposed to one. Finally, additional research is necessary to define optimal methods of developing sequential and/or cyclical combinations of PTH and antiresorptive agents. Long-term safety and efficacy of such combinations remain to be determined.

## Author Contributions

CZ and CS were involved in all aspects of the project from initial conception to writing of the manuscript.

## Funding

This work was supported by grants from the National Natural Science Foundation of China (Project nos. 81672133 and 81874010) and National High-tech R&D Program (Project no. 2015AA020304).

## Conflict of Interest

The authors declare that the research was conducted in the absence of any commercial or financial relationships that could be construed as a potential conflict of interest.
